# Epithelioid angiomyolipoma: a rare renal tumor with histopathological complexity

**DOI:** 10.1093/jscr/rjag316

**Published:** 2026-04-28

**Authors:** Mohannad N AbuHaweeleh, Abdelkareem Alhyari, Rajen Goyal, Ibrahim A Khalil, Alaeddin Badawi, Khalid Al-Rumaihi

**Affiliations:** College of Medicine, QU Health, Qatar University, PO Box 2713, Doha, Qatar; Department of Urology, Ambulatory Care Center, Hamad Medical Corporation, PO Box 3050, Doha, Qatar; Department of Laboratory Medicine and Pathology, Hamad Medical Corporation, PO Box 3050, Doha, Qatar; Department of Laboratory Medicine and Pathology, Hamad Medical Corporation, PO Box 3050, Doha, Qatar; Department of Urology, Ambulatory Care Center, Hamad Medical Corporation, PO Box 3050, Doha, Qatar; Department of Urology, Ambulatory Care Center, Hamad Medical Corporation, PO Box 3050, Doha, Qatar; Department of Urology, Ambulatory Care Center, Hamad Medical Corporation, PO Box 3050, Doha, Qatar

**Keywords:** benign renal tumor, angiomyolipoma, epitheloid angiomyolipoma, kidney tumor, uro-oncology

## Abstract

Renal angiomyolipomas (AMLs) are rare benign kidney tumors. Epithelioid angiomyolipoma (EAML) is a distinct variant with unique histopathological features and recognized malignant potential. This study highlights the diagnostic features and pitfalls of EAML. A 41-year-old woman was referred after an incidental left renal mass was detected on ultrasound. She was asymptomatic, with unremarkable examination and laboratory results, though her family history included several malignancies. Magnetic resonance imaging revealed a 3.5-cm partly exophytic, posterior lower-pole renal mass without pelvicalyceal invasion. The patient underwent robotic-assisted left partial nephrectomy. Histopathology showed a tumor composed predominantly of epithelioid cells with reduced vascularity, thick-walled vessels, and adipose tissue. Immunohistochemistry demonstrated positivity for smooth muscle actin (SMA) and Human Melanoma Black-45 (HMB-45) and negativity for Paired box 8 (PAX-8), confirming EAML. EAML poses diagnostic and management challenges due to uncertain malignant potential and lack of guidelines. Surgical resection remains primary treatment, with long-term surveillance recommended.

## Introduction

Renal angiomyolipomas (AMLs) are uncommon benign adult renal mesenchymal tumors [1] composed of blood vessels (angio-), smooth muscle (myo-), and adipose tissue (lipo-) [2]. Several subtypes of AML exist, including epithelioid, oncocytic, fat-predominant, smooth muscle-predominant, AML with epithelial cysts, and sclerosing variants [1, 3]. Epithelioid angiomyolipoma (EAML) has been defined as a rare variant of AML comprising at least 80% of epithelioid cells, according to the 2022 World Health Organization (WHO) classification of tumors of the kidney [1]. Although its global incidence is low, studies suggest that EAML shows unique biological behavior compared to classic AML [4, 5]. Currently, there are no established clinical guidelines for managing EAML, nor standardized systemic treatment protocols for metastatic cases. This gap in research leads to uncertainty regarding optimal therapeutic strategies [4]. The aim of this report is to delve deeper into the pathological aspects of EAML, exploring its distinctive histopathological features, and the challenges it presents in differential diagnosis. Hereby, we present a case of a middle-aged adult referred to our clinic due to an incidentally discovered renal mass on imaging, subsequently diagnosed as EAML through histopathology and immunohistochemistry.

## Case presentation

A 41-year-old woman, with a family history of breast, lung, and colon cancer, presented with an incidental left renal mass discovered on ultrasonography. Abdominal examination revealed no abnormalities or palpable masses. Laboratory investigations, including a complete blood count, coagulation profile, blood chemistry, endocrinology panel, and urine microscopy, were all unremarkable, as summarized in Table 1.

**Table 1:** Basic laboratory results

**Laboratory parameter**	**Result**	**Reference range**
Hgb (g/dl)	12.2	(12.0–15.0)
Hct (%)	38.7	(36.0–46.0)
Platelet (×10^3^/μl)	251	(150–410)
Urea (mmol/l)	4.6	(2.5–7.8)
Creatinine (μmol/l)	71	(44–80)
eGFR (ml/min/1.73 m^2^)	>60	>60

Hgb: hemoglobin; Hct: hematocrit; eGFR: estimated glomerular filtration rate.

## Methods and investigations

**Figure 1:**
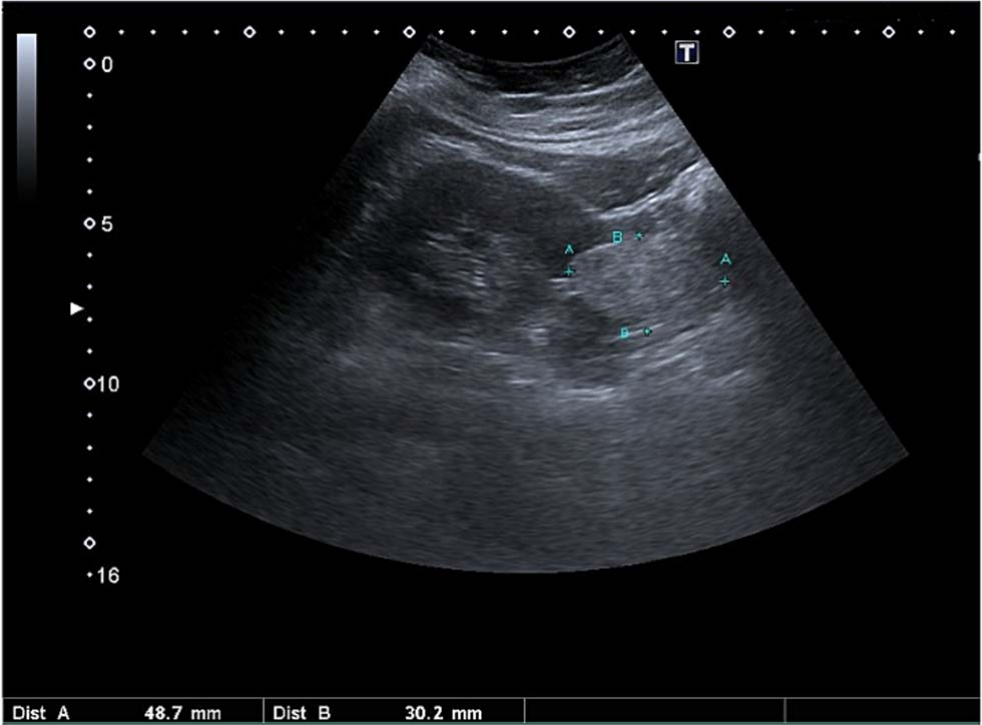
US examination of the abdomen showed a left renal mass.

**Figure 2:**
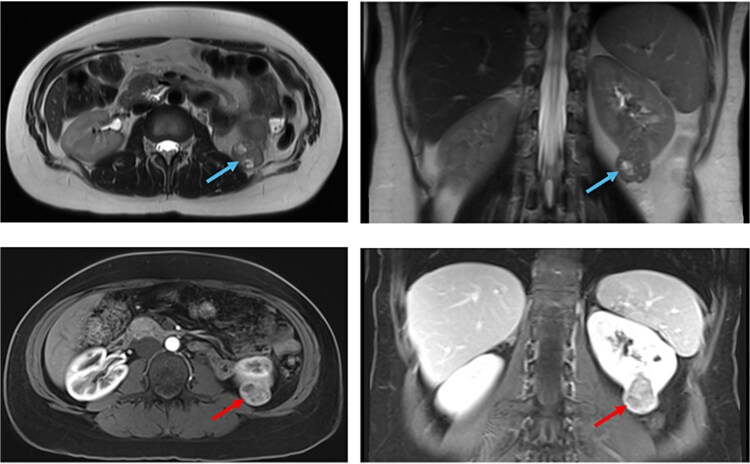
MRI examination of the abdomen showed a partially exophytic left renal mass on T2 sequence (blue arrows), and on contrasted T1 sequence (red arrows).

Initial abdominal ultrasound (US) showed a 4.8 × 3 cm mass in the lower pole of the left kidney (Fig. 1). Magnetic resonance imaging (MRI) confirmed the presence of a single lesion, measuring 3.5 cm, located in the lower pole of the left kidney, arising posteriorly, with no invasion of the pelvicalyceal system. The mass showed an intermediate T2/T1 signal with hyperintense T2/hypointense T1 cystic foci, diffusion restriction, and heterogenous enhancement (Fig. 2). The patient underwent a left robotic-assisted partial nephrectomy with an uneventful postoperative recovery.

**Figure 3:**
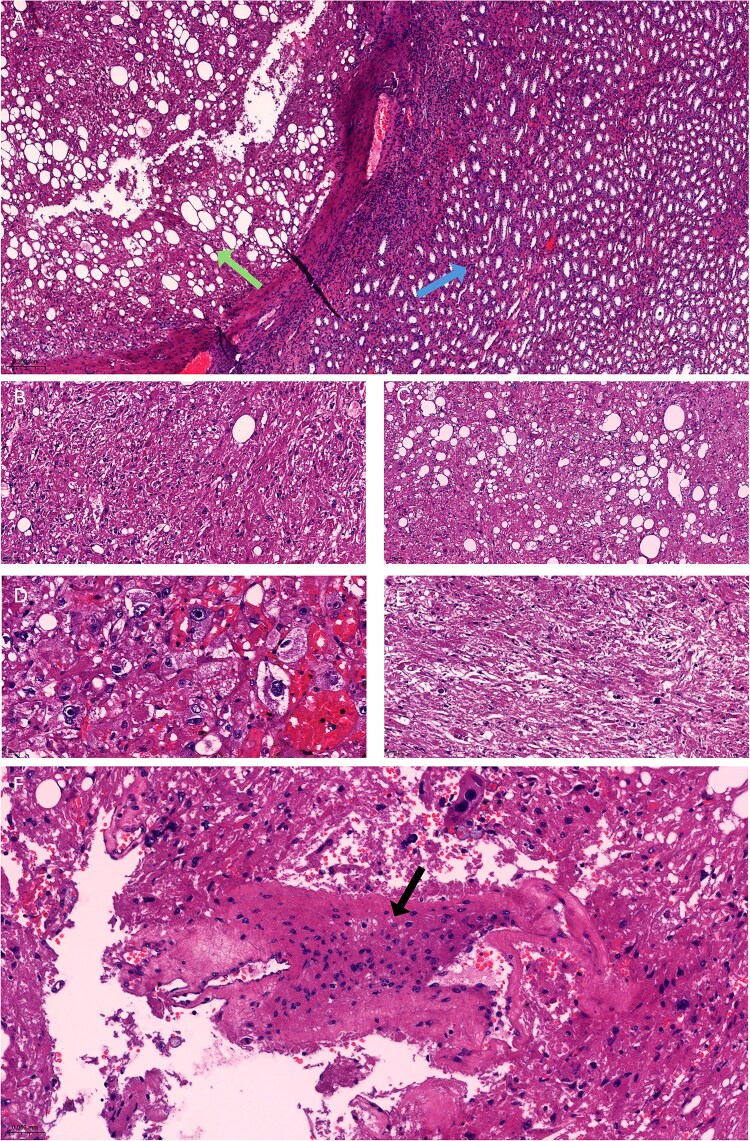
Histopathology showed (A) a well-circumscribed lesion with a capsule (green arrow) separating it from the adjacent renal parenchyma (blue arrow) (H&E, ×50). (B and C) The lesion consisted of diffuse sheets of epithelioid cells, plump spindle cells, and adipocytes (H&E, ×200). (D) Epithelioid cells with eosinophilic cytoplasm and atypical nuclei bearing prominent nucleoli, some of the epithelioid cells showed clear or finely granular cytoplasm, and mostly uniform nuclei (H&E, ×400). (E) Plump spindle cells (H&E, ×200). (F) Thickened irregular blood vessels with partial branching (black arrow) (H&E, ×200).

**Figure 4:**
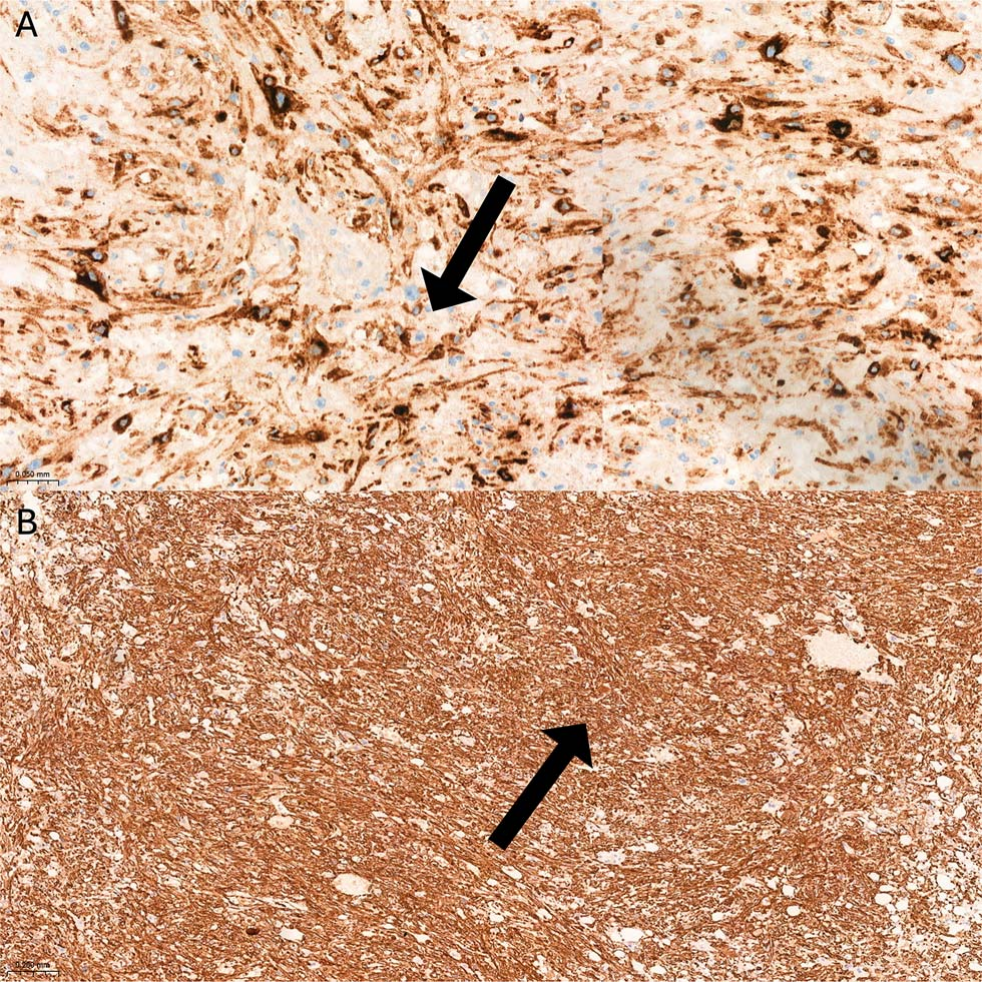
Immunohistochemical analysis showed (A) epithelioid cells demonstrated diffuse and strong cytoplasmic positivity for HMB-45, indicated by intense dark brown staining (IHC, ×200). (B) SMA showed positive cytoplasmic staining in both spindle-shaped and epithelioid smooth muscle cells, evidenced by dark brown coloration (IHC, ×40).

Gross examination revealed a 3.7 × 3.6 × 1.6 cm solid, well-demarcated, unencapsulated mass with a homogeneous tan cut surface and no necrosis. Microscopically, the tumor was well circumscribed (Fig. 3A) and composed of sheet-like nests of eosinophilic epithelioid to spindled cells. Scattered adipocytes and thick-walled irregular blood vessels were present (Fig. 3B, C, and F). Epithelioid cells comprised ~80% of the tumor with pleomorphic nuclei and prominent nucleoli (Fig. 3D). Smaller uniform epithelioid cells and plump spindle cells were also observed (Fig. 3D and E). Mitoses were rare (<1/mm^2^). Tumor cells were diffusely positive for HMB-45 (Fig. 4A) and SMA (Fig. 4B) and negative for epithelial markers, PAX-8 and S-100, confirming EAML.

The case was discussed at the postoperative multidisciplinary team meeting, and the patient was placed on structured surveillance according to low-risk renal cell carcinoma follow-up protocols, including periodic clinical evaluation and cross-sectional imaging to monitor for recurrence or progression.

## Discussion

We report a case of EAML in a previously healthy middle-aged woman who underwent left robotic-assisted partial nephrectomy. Although renal AML is a rare benign tumor, it can mimic renal cell carcinoma [6] and may lead to complications that contribute to morbidity and mortality [2].

EAML is an uncommon tumor, accounting for about 1% of renal tumors and 4.6%–7.7% of AMLs [5, 7]. While most cases are sporadic [4], over 50% are associated with autosomal dominant tuberous sclerosis complex (TSC) [5]. Mutations in tumor suppressor genes TSC1 and TSC2 disrupt the regulation of the mTORC1 pathway, resulting in increased cell growth and differentiation that contribute to the development of EAML and other TSC-related tumors [5, 8].

EAML most commonly affects middle-aged women and is often asymptomatic, frequently discovered incidentally [4, 5]. Sporadic AMLs are typically unilateral and smaller, with bleeding risk mainly when tumors become large [2]. Preoperative diagnosis of EAML is challenging, as imaging findings may resemble renal malignancy. CT is usually the primary imaging modality, while MRI can help identify fat-poor lesions. When imaging is inconclusive, biopsy or surgical intervention may be required, and uncertain masses should be managed as potential renal cancer [8]. Imaging can also assess prognostic factors such as tumor size, metastasis, lymphadenopathy, extrarenal extension, and renal vein invasion [9].

Although often considered benign, EAML has malignant potential and may behave aggressively [7]. Reported aggressiveness rates range from 5% to 66% [1]. Certain clinicopathological features are associated with aggressive behavior, including ≥70% atypical epithelioid cells, increased mitotic activity, atypical mitoses, and necrosis [10]. Additional factors linked to metastasis and poor prognosis include tumor size >70 mm, carcinoma-like growth patterns, perinephric fat or renal vein invasion, and concurrent TSC [10]. A proposed risk stratification model categorizes tumors as low (0–1 parameters), intermediate [2, 3], or high risk (≥4), with progression rates of ~15%, 64%, and 80%–100%, respectively [11]. Based on this, our case has a low risk of progression, nevertheless, scheduled for structured postoperative surveillance following low-risk renal cell carcinoma protocols [12].

Radiologically, EAMLs are typically hyperattenuating (>45 HU) on precontrast CT with heterogeneous or multilocular cystic enhancement, demonstrate T2 hypointensity with heterogeneous or multilocular cystic enhancement on MRI, and appear iso- to hypoechoic on ultrasound [13]. Histologically, EAML may demonstrate either a carcinoma-like pattern or a diffuse pattern of epithelioid and spindle cells [14]. The carcinoma-like pattern consists of large atypical eosinophilic cells with prominent nucleoli, intranuclear inclusions, and vascular septa, with occasional mitoses and necrosis [14, 15]. Immunohistochemically, EAML expresses melanocytic markers such as HMB-45, Melan-A, and microphthalmia transcription factor, along with smooth muscle markers [14, 15]. In our case, tumor cells were positive for HMB-45 and SMA and negative for epithelial markers, PAX-8 and S-100, helping exclude renal cell carcinoma and metastatic melanoma.

Currently, no specific clinical guidelines exist for the management of EAML [4]. Surgical resection remains the primary treatment, with partial nephrectomy generally recommended for tumors <4 cm and radical nephrectomy for larger tumors [5]. Selective arterial embolization or ablation may be used in patients with hemorrhage or those unfit for surgery [5]. Targeted therapies such as mTOR inhibitors (rapamycin, everolimus) have shown promise in reducing tumor size by targeting the dysregulated mTOR pathway [5, 8, 16]. Emerging evidence also suggests that PD-1/PD-L1 immunotherapy may inhibit tumor growth and improve outcomes, though further clinical research is required [5, 17].

## Conclusion

EAML is a rare renal tumor with potential malignant behavior, often difficult to distinguish from renal cell carcinoma and requiring histopathological and immunohistochemical confirmation. Surgical excision is the main treatment, but the absence of standardized guidelines highlights the need for further research into targeted, systemic, and personalized therapies.

## Consent

Written informed consent was obtained from the patient for publication of this case report and any accompanying images.

## Data Availability

Data sharing not applicable to this article as no datasets were generated or analyzed during the current study.
